# cGMP compliant one-step, one-pot automated [^18^F]FBnTP production for clinical imaging of mitochondrial activity

**DOI:** 10.1186/s41181-024-00274-y

**Published:** 2024-06-27

**Authors:** Mai Lin, Cong-Dat Pham, Robert T. Ta, H. Charles Manning

**Affiliations:** 1 Cyclotron Radiochemistry Facility, The University of Texas MD Anderson Cancer Center, Houston, TX 77054 USA; 2 Department of Cancer Systems Imaging, The University of Texas MD Anderson Cancer Center, Houston, TX 77030 USA

**Keywords:** [^18^F]FBnTP, Automation, Radiopharmaceutical, PET

## Abstract

**Background:**

4-[^18^F]fluorobenzyl-triphenylphosphonium ([^18^F]FBnTP) is a lipophilic cation PET tracer. The cellular uptake of [^18^F]FBnTP is correlated with oxidative phosphorylation by mitochondria, which has been associated with multiple critical diseases. To date, [^18^F]FBnTP has been successfully applied for imaging myocardial perfusion, assessment of severity of coronary artery stenosis, delineation of the ischemic area after transient coronary occlusion, and detection/quantification of apoptosis in various animal models. Recent preclinical and clinical studies have also expanded the possibilities of using [^18^F]FBnTP in oncological diagnosis and therapeutic monitoring. However, [^18^F]FBnTP is typically prepared through a tediously lengthy four-step, three-pot reaction and required multiple synthesizer modules; Thus, such an approach remains a challenge for this promising radiopharmaceutical to be implemented for routine clinical studies. Herein, we report an optimized one-step, one-pot automated approach to produce [^18^F]FBnTP through a single standard commercially-available radiosynthesizer that enables centralized production for clinical use.

**Results:**

The fully automated production of [^18^F]FBnTP took less than 55 min with radiochemical yields ranging from 28.33 ± 13.92% (non-decay corrected), apparent molar activity of 69.23 ± 45.62 GBq/µmol, and radiochemical purities of 99.79 ± 0.41%. The formulated [^18^F]FBnTP solution was determined to be sterile and colorless with a pH of 4.0–6.0. Our data has indicated no observable radiolysis after 8 h from the time of final product formulation and maximum assay of 7.88 GBq.

**Conclusions:**

A simplified and cGMP-compliant radiosynthesis of [^18^F]FBnTP has been established on the commercially available synthesizer in high activity concentration and radiochemical purity. While the preclinical and clinical studies using [^18^F]FBnTP PET are currently underway, the automated approaches reported herein facilitate clinical adoption of this radiotracer and warrant centralized production of [^18^F]FBnTP for imaging multiple patients.

## Background

Mitochondria are recgonized as the power plants of the cell and the organelles where 90% of ATP are produced (Pizzorno 2014). Since mitochondria are also involved with metabolism, ion homeostasis, and signaling pathways during different stages of the cell cycle, a long list of common diseases such as cancer, diabetes, neurodegenerative and cardiovascular disorders is now believed to be caused by or aggravated by mitochondrial dysfunction (Javadov et al. 2020). However, performing a direct and dynamic evaluation of patients with abnormal mitochondrial functionality remains challenging.

Nuclear imaging can be a powerful tool to overcome such challenge and monitor the metabolic changes in patients with mitochondrial diseases. Among all techniques that have been developed so far, monitoring mitochondrial membrane potential (ΔΨm) with lipophilic, cationic dyes has emerged as one of the most used methodologies (Ehrenberg et al. 1988). Lipophilic cations can penetrate biological membranes by passive diffusion into the cytoplasm and mitochondria due to large negative plasma and mitochondrial membrane potentials (ref.). [^99m^Tc]Tc-Sestamibi, [^99m^Tc]Tc-Tetrofosmin, and [^99m^Tc]Tc-Teboroxime are the only lipophilic cations that have been approved by the FDA for single-photon emission computed tomography (SPECT) imaging (Boschi et al. 2022). However, the clinical use of [^99m^Tc]Tc-Teboroxime has quickly declined in clinical practice due to its rapid myocardial washout (Boschi et al. 2022). To date, only [^99m^Tc]Tc-Sestamibi and [^99m^Tc]Tc-Tetrofosmin are routinely used as myocardial-perfusion as well as tumor imaging agents (Boschi et al. 2022; Schillaci, et al. 2013; Treglia, et al. 2007; Spanu, et al. 2005; Schillaci, et al. 2005).

In addition to SPECT, positron emission tomography (PET) is another popular nuclear imaging techniques for routine clinical practice. Compared to SPECT, PET exhibits superior sensitivity and relatively straight forward imaging quantification process after corrections for photon attenuation and scattering (Rahmim and Zaidi 2008). Following the success of [^99m^Tc]Tc-Sestamibi and [^99m^Tc]Tc-Tetrofosmin, triphenylmethylphosphonium-based PET radiotracers have gradually demonstrated their potentials to monitor mitochondrial functionality through visualizing its membrane potential. ^11^C-Labeled triphenylmethylphosphonium ([^11^C]TPMP) is the first triphenylmethylphosphonium-based PET radiotracer and its encouraging outcome for myocardial and tumor imaging has been well recognized (Krause et al. 1994). However, the clinical adaption of [^11^C]TPMP is hindered by the short half-life of ^11^C (T_1/2_: 20 min). As a consequence, 4-[^18^F]fluorobenzyl-triphenylphosphonium ([^18^F]FBnTP) (T_1/2_ of ^18^F: 109.7 min) has been developed as a next generation of the PET radiotracer to target mitochondrial activity. [^18^F]FBnTP has shown great promise in various animal models with mitochondrial metabolic disease (Madar et al. 2009, 2011, 2006; Higuchi et al. 2011; Momcilovic et al. 2019) and is currently under a clinical trial (NCT02204462).

Producing radiotracers with highly consistent radiochemical yield and quality is required to bring promising radiotracers from bench to bedside. [^18^F]FBnTP was originally reported to be synthesized through a four-step, three-pot reaction (Ravert et al. 2004a). Although the overall number of reaction steps was reduced to three (Ravert et al. 2014; Tominaga et al. 2016), such multi-step approach remains a challenge for implementing the radiosynthesis of [^18^F]FBnTP for routine clinical practices. According to Zhang et al., it is feasible to prepare [^18^F]FBnTP in one step via a copper‐mediated [^18^F]fluorination reaction (Zhang et al. 2016). Adopting their encouraging finding and improving upon the process, we report a detailed and highly robust technical protocol to produce the cGMP-compliant radiosynthesis of [^18^F]FBnTP using GE TRACERlab™ FXFN, the most widely used synthesis module for routine clinical radiopharmaceutical production. The protocol detailed herein greatly simplifies the production process and can be easily followed by anyone skilled in the art and equipped with common resources.

## Materials and methods

### General

The automated radiosynthesis of [^18^F]FBnTP via the GE TRACERlab™ FXFN was performed inside the aseptically cleaned COMECER hot cell under cGMP condition. Triphenyl(4‐(4,4,5,5‐tetramethyl‐1,3,2‐dioxaborolan‐2‐yl)benzyl)phosphonium bromide, silver trifluoromethanesulfonate (AgOTf), anhydrous acetonitrile (99.8%), N,N-dimethylformamide (DMF), 1,3-dimethyl-2-imidazolidinone (DMI), potassium carbonate, potassium trifluoromethanesulfonate, and tetrakis(pyridine)copper(II) triflate were purchased from MilliporeSigma (St. Louis, MO). The Alumina N Plus Light Cartridge (Part # WAT023561) and tC18 Plus Short Cartridge (Part # WAT036810) were acquired through Waters (Milford, MA). The ethanol 200 PROOF was obtained from Pharmco. The sterilized water for injection (SWI) and normal saline were the product of Baxter International (Deerfield, IL). All other references of ‘water’ refers to Milli-Q water (18 MΩ•cm) taken from a Millipore Milli-Q Integral 5 water purification system and were primarily used in quality control processes. Anhydrous acetonitrile (99.8%) used in the evaporation step was from MilliporeSigma. Nitrogen and argon gas used primarily in drying and transferring of solutions were provided through Matheson Tri-gas. The automation synthesis on the TRACERlab™ FXFN module was controlled by the TRACERLab FX software.

### Synthesis of triphenyl(4‐(4,4,5,5‐tetramethyl‐1,3,2‐dioxaborolan‐2‐yl)benzyl)phosphonium trifluoromethanesulfonate (Fig. 1)

**Fig. 1:**
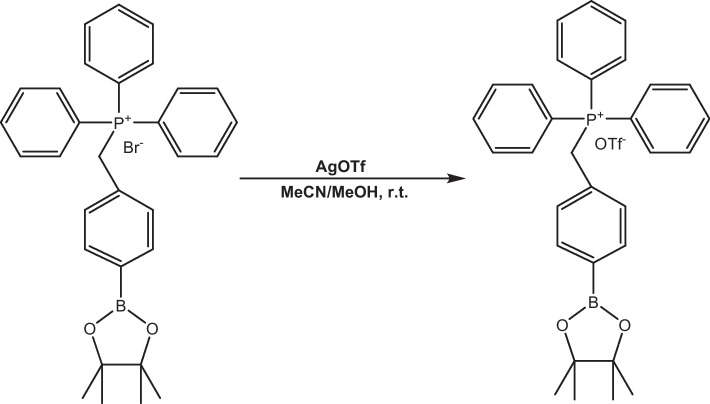
Synthesis scheme of triphenyl(4‐(4,4,5,5‐tetramethyl‐1,3,2‐dioxaborolan‐2‐yl)benzyl)phosphonium trifluoromethanesulfonate

To a solution of triphenyl(4-(4,4,5,5-tetramethyl-1,3,2-dioxaborolan-2-yl)benzyl)phosphonium bromide (1.12 g, 2 mmol) in 12 mL acetonitrile and 12 mL methanol was added AgOTf (0.51 g, 2 mmol) in 3 mL acetonitrile dropwise. The reaction mixture was stirred in the dark for 24 h. The resulted suspension was filtered through celite, and the solid was washed with methanol (2 × 5 mL). The combined filtrate was evaporated to remove volatile solvents. The residue was dissolved in 50 mL dichloromethane and filtered, evaporated under reduced pressure, and lyophilized overnight to obtain the title compound as a white solid (1.25 g, 99%). 1H NMR (300 MHz, CDCl3): δ 7.52 (m, 3H), 7.41–7.27 (m, 15H), 6.67 (dd, J1 = 2.38 Hz, J2 = 8.06 Hz, 2H), 4.79 (d, J = 15.6 Hz, 2H), 1.06 (s, 12H). F NMR (282 MHz, CDCl3): δ − 78.16. MS (ESI^+^): m/z = 479.4 [M + H]^+^

### Automated synthesis of [^18^F]FBnTP

**Fig. 2:**
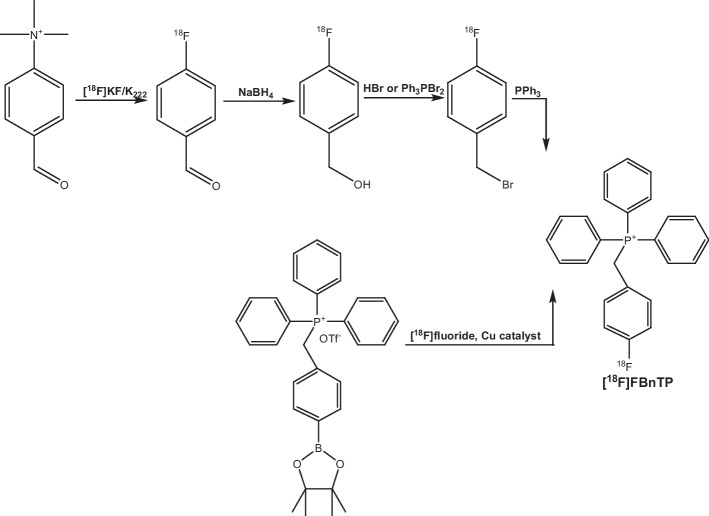
Comparative radiosynthesis scheme of preparing [^18^F]FBnTP through the original four-step, two-pot reaction and one-step, one-pot reaction performed in this work

Figure 2 illustrates the radiosynthesis scheme of preparing [^18^F]FBnTP. [^18^F]Fluoride was produced by irradiating 2.5 mL of enriched [^18^O]H_2_O with 60 μAh beam current from the 16.5 MeV GE PETrace cyclotron. The [^18^F]Fluoride was separated from the [^18^O]H_2_O by trapping the [^18^F]Fluoride on the preconditioned QMA light cartridge. Following the trapping step, 0.8 mL of the solution mixture (H_2_O/acetonitrile = 1/1) containing 5 mg of potassium triflate and 50 µg of potassium carbonate was used to elute the [^18^F]fluoride from the cartridge into the reaction vessel. The solution was heated to 120 °C to remove the water and acetonitrile initially. Next, the precursor and catalyst for the reaction, triphenyl(4‐(4,4,5,5‐tetramethyl‐1,3,2‐dioxaborolan‐2‐yl)benzyl)phosphonium trifluoromethanesulfonate and tetrakis(pyridine)copper(II) triflate dissolved in either anhydrous N,N-dimethylformamide or 1,3-dimethyl-2-imidazolidinone, were added to the reaction vessel. The reaction was heated at 110 °C for 10–20 min in a closed reaction vessel to promote the substitution of the ^18^F for the Bpin leaving group. After the radiolabeling is completed, 2 mL of water was added into the reaction vessel to dilute the reaction mixture. The solution is then passed through a pre-conditioned Alumina N Plus Light cartridge and the eluent is injected to the semi-prep HPLC for purification. Purification was performed using a Phenomenex Luna C18 column (250 mm × 4.6 mm, 5 um) and a mixture of 50% acetonitrile: 50% 50 mM ammonium acetate solution at 5 mL/min. The product fraction was collected and diluted with SWI. This solution was passed through a Sep-Pak Plus Short tC18 cartridge to retain the desired compound followed by a rinse step with SWI to remove excess acetonitrile and ammonium acetate. The final product, [^18^F]FBnTP was then eluted by 1.0 mL of ethanol and reformulated with 10 mL of saline. The solution was passed through a 0.22 µm vented sterilizing filter into a sterile vial for QC sampling and dose dispensing.

**Fig. 3:**
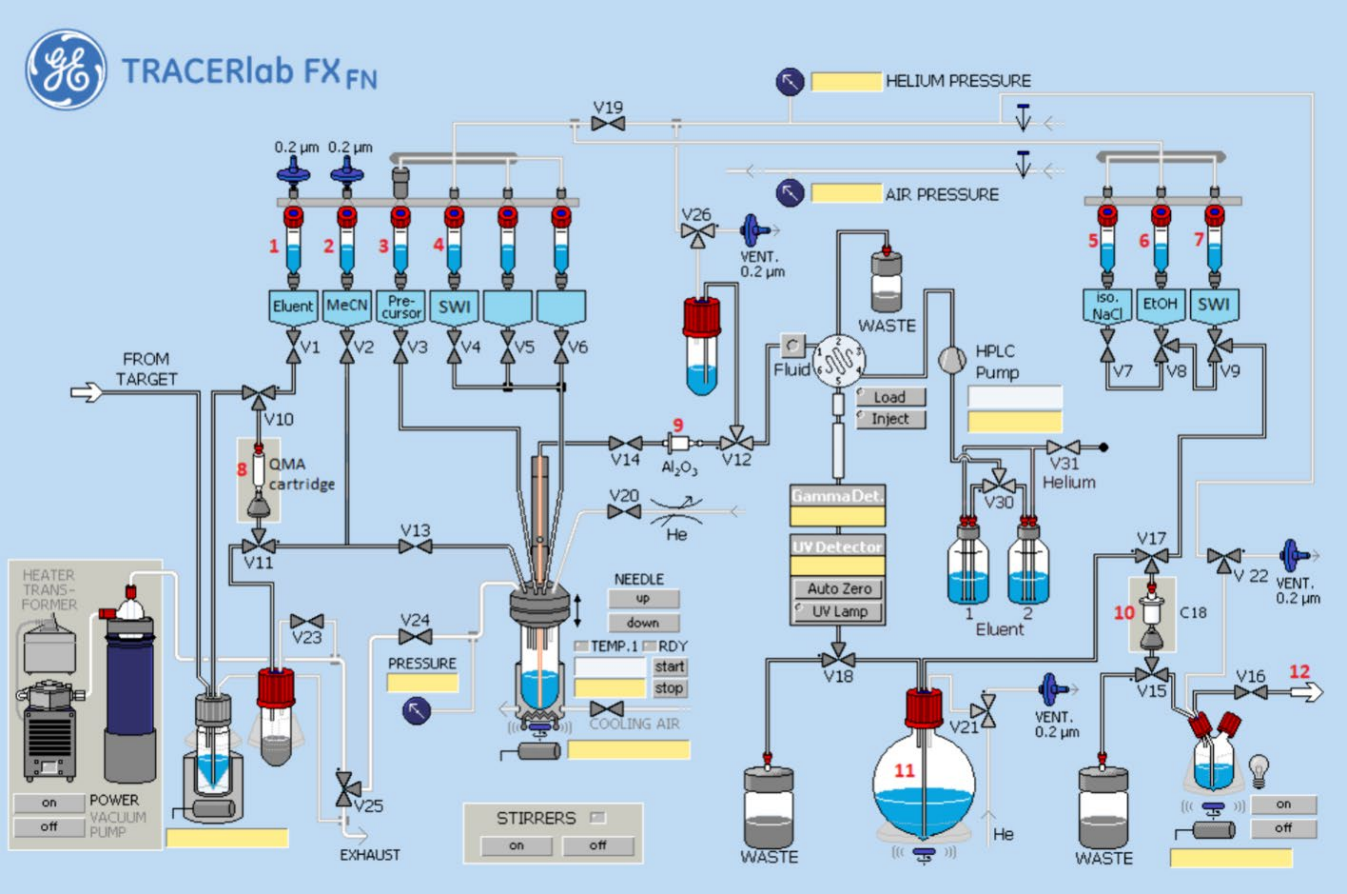
Schematic overview of the [^18^F]FBnTP radiosynthesis on the GE TRACERlab™ FXFN module. The numbers denoted in RED, represented the designation of item and position of reagents and consumables indicated on Table 1

Figure 3 illustrates the preparation setup for the radiosynthesis of [^18^F]FBnTP for injection. Vials 1 through 9 were loaded with the respective reagents shown on Table 1. At position 8, a pre-conditioned QMA cartridge was installed and used as purchased. An Alumina N Plus Light Cartridge was conditioned with 10 mL of water followed by 10 mL of air and connected to the lines at position 9. At position 10, a tC18 Plus Short cartridge that was conditioned with 10 mL of ethanol and 10 mL of water followed by 10 mL of air was installed. The HPLC collection flask containing 50 mL of water was placed at position 11.

**Table 1:** Material and reagent list used in the radiosynthesis of [^18^F]FBnTP via TRACERlab™ FXFN module

^*a*^ Item #	Reagents or consumables
1	Potassium Triflate/Potassium Carbonate QMA Elution Solution, 0.8 mL
2	Anhydrous Acetonitrile, 0.6 mL
3	FBnTP precursor, 10 mg, and Tetrakis(pyridine)copper(II) triflate, 34 mg dissolved in 1.0 mL anhydrous N,N-dimethylformamide (DMF) or 1,3-dimethyl-2-imidazolidinone (DMI)
4	Sterilized water for injection (SWI), 2.0 mL
5	Ascorbic acid, 10 mg dissolved in 10 mL normal saline
6	Ethanol, 1.0 mL
7	Sterile water for injection (SWI), 10 mL
8	Pre-conditioned QMA light Sep-Pak cartridge, 1 cartridge
9	Sep-Pak Alumina N Plus Light cartridge, 1 cartridge
10	Sep-Pak tC18 Plus Short cartridge, 1 cartridge
11	Ascorbic acid, 10 mg dissolved in sterilized water for injection (SWI), 50 mL
12	Final Product Vial, 1 vial

### Quality control

The characterization of [^18^F]FBnTP for injection was performed on the Agilent 1260 HPLC system equipped with a variable wavelength detector and Lablogic NaI radio-detector. The analytical method for determining the identity, chemical and radiochemical purity of the [^18^F]FBnTP include the following conditions: The analytical peak separation flow rate was set to 1.0 mL/min through a Phenomenex Luna C-18(2) column (150 mm × 4.6 mm × 5 μm) and the UV detector @ 254 nm. Mobile Phase (A) Water with 0.01% trifluoroacetic acid and Mobile Phase (B) Acetonitrile with 0.01% trifluoroacetic acid, with isocratic hold at 60% A (2 min) and 40% B. In this condition, [^18^F]FBnTP was eluted at around 7.5 min.

To determine the working range amount of “cold” FBnTP present in the [^18^F]FBnTP for injection, HPLC analysis of the reference standard atthe concentration levels of 12.5, 25.0, 50.0, 100.0, 250.0, and 500.0 µg/mL was conducted to establish the “best-fit” calibration curve. Thus, when analyzing [^18^F]FBnTP product during routine production, the amount of “cold” counterpart was calculated against a single point calibration method using a working reference standard (4 mg of FBnTP standard was dissolved with 10 mL of the HPLC solution), at 20µL injection volume. The residual solvents in [^18^F]FBnTP for injection was determined by Agilent GC system with FID, equipped with GC Column DB200 (30 m × 0.250 mm, 0.50 µm stationary phase thickness). The system was calibrated to determine the concentration of ethanol, isopropanol, acetonitrile, and 1,3-Dimethyl-2-imidazolidinone. The calibration curve generated from this test were subsequently used for quantification of residual solvents in the validated production batches, The radionuclidic identity and purity, residual Kryptofix® [_2.2.2_], bacterial endotoxin, sterility. appearance, pH, and filter tests were also performed under the USP < 823 > guidelines.

## Results

### Automated synthesis of [^18^F]FBnTP

**Fig. 4:**
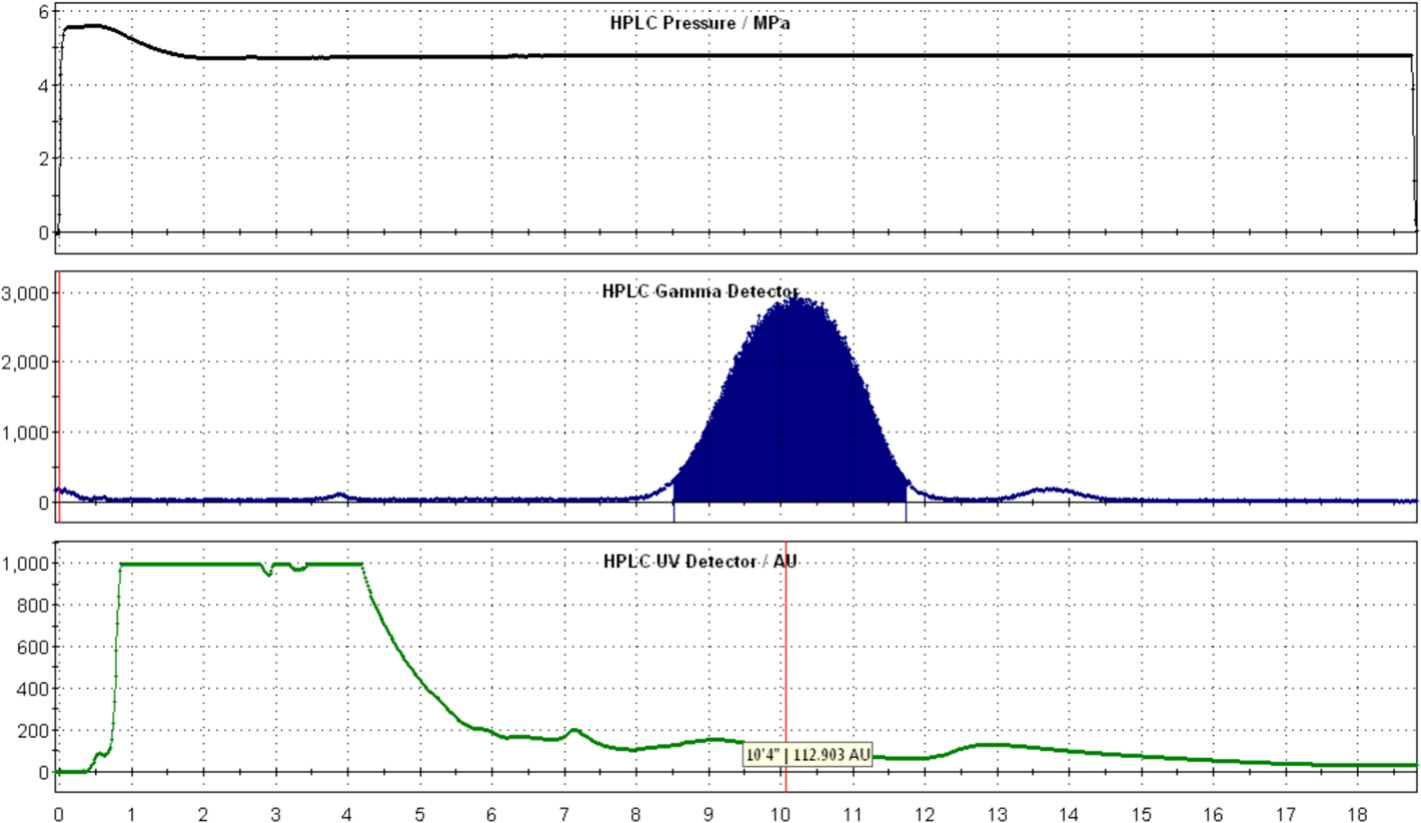
The chromatography of the [^18^F]FBnTP production during the HPLC purification process. Purification was performed using a Phenomenex Luna C18 column (250 mm × 4.6 mm, 5 um) and a mixture of 50% acetonitrile: 50% 50 mM ammonium acetate solution at 5 mL/min. The desired product was collected between 8.5 and 11.5 min

The production of [^18^F]FBnTP was able to be completed less than 1 h in the TRACERlab™ FXFN including the trapping of [^18^F]fluoride from the cyclotron to the QMA cartridge on the synthesizer. The results of [^18^F]FBnTP synthesis using the TRACERlab are shown in Table 2, in which the fraction between 8.5 and 11.5 min was typically collected during the HPLC purification process (Fig. 4). In an experiment to compare solvent suitability for [^18^F]FBnTP radiolabeling reaction, we observed that productions with starting activity in range of 37–65 GBq using N,N-dimethylformamide (DMF) as the solvent resulted in the average non-decay-corrected radiochemical yield of 6.18 ± 0.99% with radiochemical purity of 98.34 ± 1.15%. On the other hand, using 1,3-Dimethyl-2-imidazolidinone (DMI) as the reaction solvent greatly improved the ^18^F labeling efficacy, leading to the much higher radiochemical yield of 28.33 ± 13.92% and radiochemical purity of 99.79 ± 0.41%. The replacement to DMI solvent has also dramatically cut reaction time by half, from 20 to 10 min, which led to the reduction of overall production time to within 55 min. In addition, the apparent molar activity was significantly increased from 7.03 ± 2.11 GBq/µmol to 69.23 ± 45.62 GBq/µmol when switching 1,3-Dimethyl-2-imidazolidinone as the reaction solvent.

**Table 2:** Radiosynthesis of [^18^F]FBnTP via TRACERlab™ FXFN module

Entry	Precursor Amount (mg)	Catalyst Amount (mg)	Labeling Solvent*	Labeling Time (min)	Starting Activity (GBq)	[^18^F]FBnTP Product Activity @ EOS	Radiochemical Purity (%)	Non-decay Corrected Radiochemical Yield (%)
1	10	34	DMF	20	40.70	2.05	97.48	5.04
2	10	34	DMF	20	37.00	2.31	97.22	6.24
3	10	34	DMF	20	48.10	3.58	99.50	7.44
4	10	34	DMF	20	62.90	3.77	99.15	5.99
5	10	34	DMI	20	7.40	1.96	100.00	26.49
6	10	34	DMI	10	8.51	4.13	100.00	48.53
7	10	34	DMI	10	37.00	7.4	99.17	20.00
8	10	34	DMI	10	25.90	4.74	100.00	18.30
9	5	17	DMI	10	22.20	1.52	100.00	6.84
10	10	34	DMI	10	66.66	7.84	100.00	11.18
11	10	34	DMI	10	59.20	7.88	100.00	13.31

### Quality control of [^18^F]FBnTP for injection

**Fig. 5:**
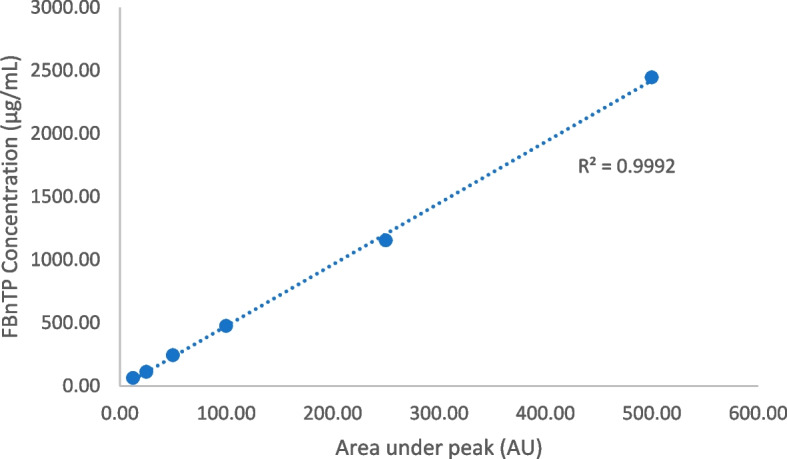
The calibration curve of FBnTP reference standard by the HPLC analysis

**Fig. 6:**
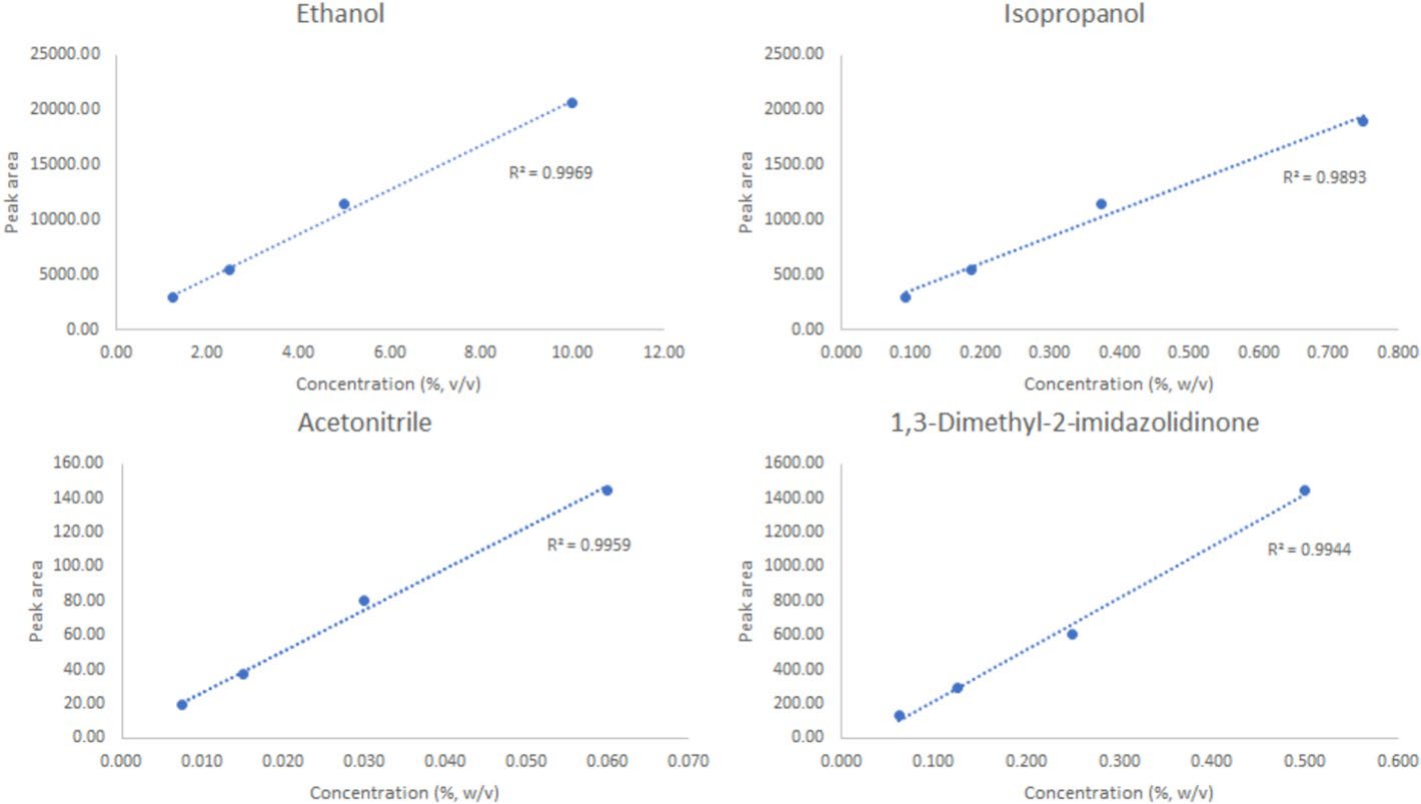
The calibration curve of ethanol, isopropanol, acetonitrile, and 1,3-Dimethyl-2-imidazolidinone by the GC analysis

**Fig. 7:**
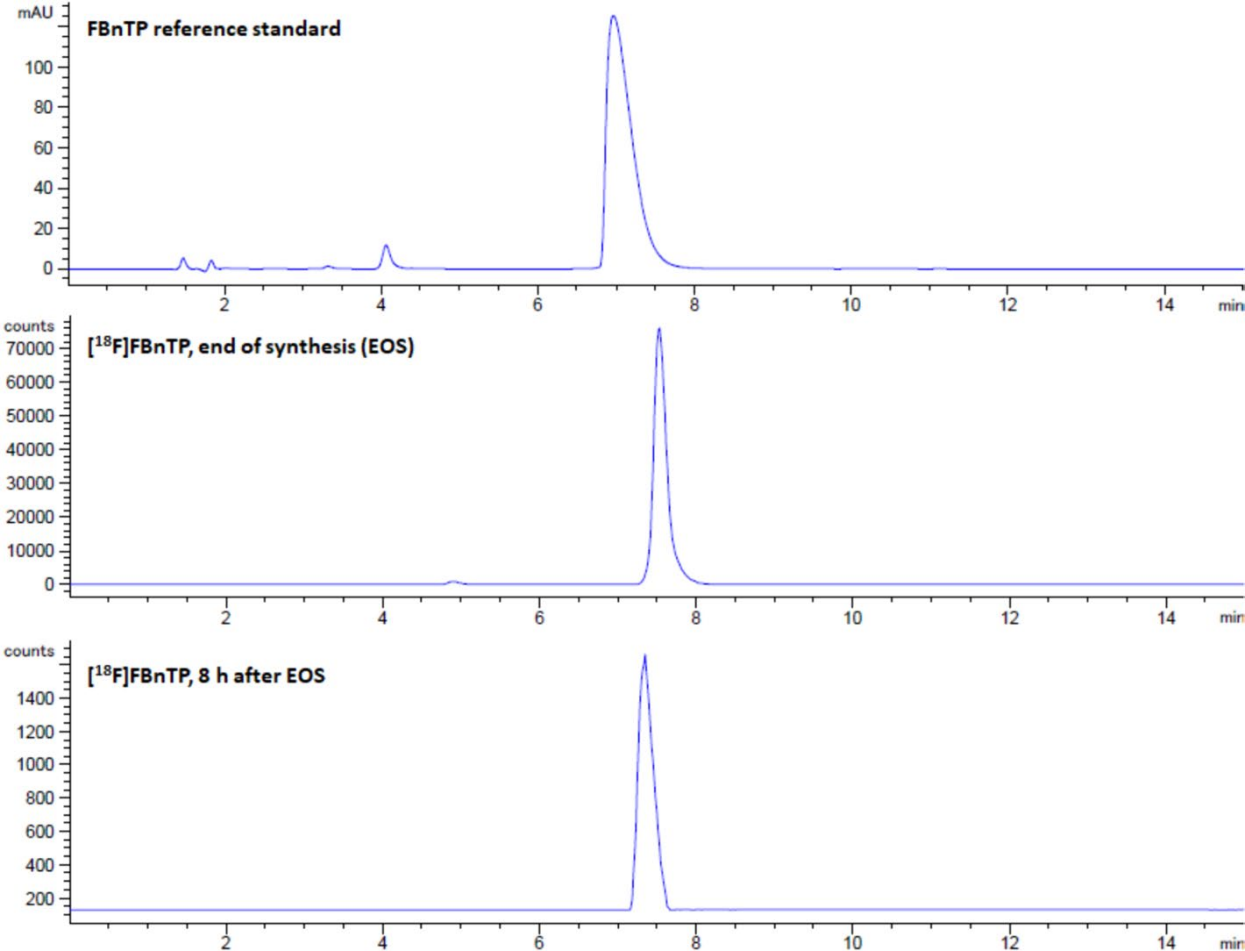
The HPLC analyses of [^18^F]FBnTP at the end of synthesis (EOS) and after 8 h of the EOS. No radiolysis was observed, and the product remained stable

**Fig. 8:**
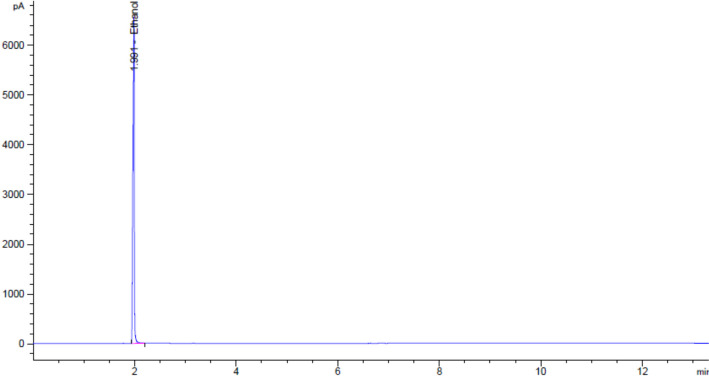
The representative GC analyses of [^18^F]FBnTP at the end of synthesis from a production batch. While the ethanol concentration was found to be 5.65% (v/v) in this batch, isopropanol, acetonitrile and 1,3-dimethyl-2-imidazolidinone were not detected

The product specifications are listed in Table 3. Figures 5 demonstrated the data results by HPLC analysis for calibration of FBnTP reference standard and Figs. 6 illustrated the data results by GC analysis for calibration of ethanol, isopropanol, acetonitrile, and 1,3-Dimethyl-2-imidazolidinone, respectively. The radiochemical purity and apparent molar activity are described in the previous section. Further HPLC analysis has confirmed that the product remained stable for 8 h post end-of-production (Fig. 7). For GC residual solvents analysis, the observedethanol concentrations ranged between 5 and 9% (v/v), whereas the concentration of acetonitrile, isoproponal, and 1,3-Dimethyl-2-imidazolidinone were negligible (Fig. 8). The pH was between 4.0 and 6.0 and the bubble point was between 70 and 75 psi. In addition, the sterility, pyrogenicity, and all other quality control tests passed specifications based on the USP < 823 > guidelines. Validation data of 3 consecutive [^18^F]FBnTP production batches are illustrated in Table 3.

**Table 3:** Product specifications of the 3 consecutive [^18^F]FBnTP production batches

Quality control tests	Specifications	Procedure	Validation 1	Validation 2	Validation 3
Filter integrity	Following manufacture’s specification	Bobble point test	72 psi	72 psi	72 psi
Appearance	Clear, colorless, particle-free	Visual observation	Pass	Pass	Pass
pH	4.0–7.0	Narrow range pH paper	5.0	4.5	4.5
Strength	74–925 MBq/mL at EOS	Capintec dose calibrator	672.72 MBq/mL	430.91 MBq/mL	712.72 MBq/mL
Radiochemical purity	≥ 90%	HPCL analysis	99.17%	100%	100%
Radiochemical identity	Retention time of [^18^F] test sample should be within ± 10% of the cold standard retention time	HPCL analysis	Pass (8.03%)	Pass (8.08%)	Pass (6.51%)
Chemical purity by GC	Solvent: Ethanol ≤ 10.0%v/v	GC analysis	8.37%	7.92%	5.65%
	Residual solvent: Acetonitrile ≤ 0.041% w/v	GC analysis	Not detected	Not detected	Not detected
	Residual solvent: Isopropanal ≤ 0.5% w/v	GC analysis	Not detected	Not detected	Not detected
	Residual solvent: 1,3 Dimethyli-2-imidazolidinone ≤ 0.041% w/v	GC analysis	Not detected	Not detected	Not detected
Kryptofix test	≤ 50 μg/mL	Color spot test	Pass	Pass	Pass
Bacterial endotoxin	≤ 17.5 Eu/mL	Bacterial endotoxin/LAL test	< 2.5 EU/mL	< 2.5 EU/mL	< 2.5 EU/mL
Radionuclidic identity by half life	105–115 min	Capintec dose calibrator	110 min	110 min	111 min
Radionuclidic identity	≥ 99.5%	Caprac well counter analysis	Pass	Pass	Pass
Sterility	No growth observed in 14 days	Visual observation	Pass	Pass	Pass

## Discussion

[^18^F]FBnTP is a promising PET tracer to target negative membrane potential, but its routine application in the clinic is hampered by the complicated multistep radiosynthesis. In this study, a robust synthesis protocol for the multi-dose production of [^18^F]FBnTP through a one-step, one-pot automated approach was successfully established on the GE TRACERlab™ FXFN radiosynthesizer.

[^18^F]FBnTP was initially reported to be synthesized through four-step, two-pot reactions that involves fluorination, reduction, bromination, and coupling with triphenylphosphine (Ravert et al. 2004b). A microwave-facilitated synthesis using custom hardware was later reported by the same research group (Ravert et al. 2014), and a relatively simplified three-step, two-pot reactions were further developed (Tominaga et al. 2016). Although the original four-step synthesis has been translated into an automated approach by Waldmann et al. (2018), such strategy remains a challenge for implementing the synthesis of [^18^F]FBnTP for routine clinical studies as most automated synthetic modules are equipped with only one reactor.

With the advancement of aromatic nucleophilic substitution through copper-mediated fluorination, Zhang et al. revealed the possibility to produce [^18^F]FBnTP using one-step, one-pot reaction (Zhang et al. 2016). However, the synthesis was performed manually and neither radiochemical yield nor molar activity of the purified product was reported. Resuming this possibility for translation into clinical settings, we have further developed and optimized the automated approach to produce [^18^F]FBnTP that is cGMP compliant. We initially followed the conditions reported by Zhang et al. (2016) using TRACERlab with higher starting activity (2.59–11.1 GBq). In contrast to the authors’ observation, we found that using tetrakis(pyridine)copper(II) triflate as the catalyst provided a higher radiolabeling efficiency in our automated module, and the formation of tetrakis(pyridine)copper(II) was not as efficiently when copper(II) triflate and pyridine were individually added for reaction. Nevertheless, the radiochemistry yields were still relatively low (6.18 ± 0.99%) and not well suited for radiotracer production on a large scale. Recently, Hoffmann et al. found 1,3-dimethyl-2-imidazolidinone to be an ideal solvent to promote copper-mediated fluorination (Hoffmann et al. 2023). The group systematically evaluated a series of complexes and identified several applicable mediators for highly efficient radiofluorination of aryl boronic and stannyl substrates. Utilization of these mediators in n-butanol/ 1,3-Dimethyl-2-imidazolidinone mixture or 1,3-Dimethyl-2-imidazolidinone alone significantly improved ^18^F‐labeling yields. In agreement with their findings, we have observed that 1,3-dimethyl-2-imidazolidinone not only significantly improved the radiochemical yield of [^18^F]FBnTP from 6.18 ± 0.99% to 28.33 ± 13.92%, but also reduced the labeling time from 20 to 10 min. However, in contrast to being able to maintain the higher ^18^F‐labeling yields using lower precursor amounts by Hoffmann et al., we observed reduced production yields in our case. Compared to the previous reporting of [^18^F]FBnTP production that requires 90–92 min on the ELIXYS FLEX/CHEM radiosynthesizer (Waldmann et al. 2018), our production time was greatly reduced to within 55 min. More importantly, all commercially available cartridges can be used directly without any modifications in our production process, resulting in an protocol for cGMP manufacturing of [^18^F]FBnTP for injection.

## Conclusions

CGMP-compliant automated radiosynthesis and quality control of [^18^F]FBnTP have been established on a standard commercially available single reactor synthesizer with high activity concentration, apparent molar activity, and radiochemical purity. While the preclinical and clinical trials using [^18^F]FBnTP PET are currently underway, the approach reported herein mitigate the clinical adoption of this radiotracer and warrant centralized and large-scale production of [^18^F]FBnTP.

## Data Availability

All data generated or analyzed during this study are included in this manuscript.

## References

[CR1] Boschi A, Uccelli L, Marvelli L, Cittanti C, Giganti M, Martini P (2022). Technetium-99m radiopharmaceuticals for ideal myocardial perfusion imaging: lost and found opportunities. Molecules.

[CR2] Ehrenberg B, Montana V, Wei MD, Wuskell JP, Loew LM (1988). Membrane potential can be determined in individual cells from the nernstian distribution of cationic dyes. Biophys J.

[CR3] Higuchi T, Fukushima K, Rischpler C, Isoda T, Javadi MS, Ravert H, Holt DP, Dannals RF, Madar I, Bengel FM (2011). Stable delineation of the ischemic area by the pet perfusion tracer ^18^f-fluorobenzyl triphenyl phosphonium after transient coronary occlusion. J Nucl Med.

[CR4] Hoffmann C, Kolks N, Smets D, Haseloer A, Gröner B, Urusova EA, Endepols H, Neumaier F, Ruschewitz U, Klein A, Neumaier B, Zlatopolskiy BD (2023). Next generation copper mediators for the efficient production of (18) F-labeled aromatics. Chemistry.

[CR5] Javadov S, Kozlov AV, Camara AKS (2020). Mitochondria in health and diseases. Cells.

[CR6] Krause BJ, Szabo Z, Becker LC, Dannals RF, Scheffel U, Seki C, Ravert HT, Dipaola AF, Wagner HN (1994). Myocardial perfusion with [11c]methyl triphenyl phosphonium: measurements of the extraction fraction and myocardial uptake. J Nucl Biol Med.

[CR7] Madar I, Huang Y, Ravert H, Dalrymple SL, Davidson NE, Isaacs JT, Dannals RF, Frost JJ (2009). Detection and quantification of the evolution dynamics of apoptosis using the pet voltage sensor 18f-fluorobenzyl triphenyl phosphonium. J Nucl Med.

[CR8] Madar I, Isoda T, Finley P, Angle J, Wahl R (2011). ^18^f-fluorobenzyl triphenyl phosphonium: a noninvasive sensor of brown adipose tissue thermogenesis. J Nucl Med.

[CR9] Madar I, Ravert HT, Du Y, Hilton J, Volokh L, Dannals RF, Frost JJ, Hare JM (2006). Characterization of uptake of the new pet imaging compound ^18^f-fluorobenzyl triphenyl phosphonium in dog myocardium. J Nucl Med.

[CR10] Momcilovic M, Jones A, Bailey ST, Waldmann CM, Li R, Lee JT, Abdelhady G, Gomez A, Holloway T, Schmid E, Stout D, Fishbein MC, Stiles L, Dabir DV, Dubinett SM, Christofk H, Shirihai O, Koehler CM, Sadeghi S, Shackelford DB (2019). In vivo imaging of mitochondrial membrane potential in non-small-cell lung cancer. Nature.

[CR11] Pizzorno J (2014). Mitochondria-fundamental to life and health. Integr Med (encinitas).

[CR12] Rahmim A, Zaidi H (2008). Pet versus spect: strengths, limitations and challenges. Nucl Med Commun.

[CR13] Ravert HT, Holt DP, Dannals RF (2014). A microwave radiosynthesis of the 4-[18f]-fluorobenzyltriphenylphosphonium ion. J Labelled Comp Radiopharm.

[CR14] Ravert H, Madar I, Dannals R (2004). Radiosynthesis of 3-[^18^f]fluoropropyl and 4-[^18^f]fluorobenzyl triarylphosphonium ions. J Labelled Compd Radiopharm.

[CR15] Schillaci O, Spanu A, Danieli R, Madeddu G (2013). Molecular breast imaging with gamma emitters. Q J Nucl Med Mol Imaging.

[CR16] Schillaci O, Spanu A, Madeddu G (2005). [^99m^tc]sestamibi and [^99m^tc]tetrofosmin in oncology: spet and fusion imaging in lung cancer, malignant lymphomas and brain tumors. Q J Nucl Med Mol Imaging.

[CR17] Spanu A, Schillaci O, Madeddu G (2005). ^99m^tc labelled cationic lipophilic complexes in malignant and benign tumors: the role of spet and pinhole-spet in breast cancer, differentiated thyroid carcinoma and hyperparathyroidism. Q J Nucl Med Mol Imaging.

[CR18] Tominaga T, Ito H, Ishikawa Y, Iwata R, Ishiwata K, Furumoto S (2016). Radiosynthesis and preliminary biological evaluation of a new (18)F-labeled triethylene glycol derivative of triphenylphosphonium. J Labelled Comp Radiopharm.

[CR19] Treglia G, Spitilli MG, Calcagni ML, Giordano A (2007). The role of nuclear medicine in the management of thymomas. Ann Ital Chir.

[CR20] Waldmann CM, Gomez A, Marchis P, Bailey ST, Momcilovic M, Jones AE, Shackelford DB, Sadeghi S (2018). An automated multidose synthesis of the potentiometric pet probe 4-[(18)F]fluorobenzyl-triphenylphosphonium ([(18)F]fbntp). Mol Imaging Biol.

[CR21] Zhang Z, Zhang C, Lau J, Colpo N, Bénard F, Lin K-S (2016). One-step synthesis of 4-[^18^f]fluorobenzyltriphenylphosphonium cation for imaging with positron emission tomography. J Labelled Compd Radiopharm.

